# Around the Hospitals

**Published:** 1895-12-14

**Authors:** 


### Around the Hospitals.

[Notice to the Managers of Institutions.—Special space is now reserved for the insertion of notices of hospital meetings and festivals. The Editor requests that all notices may reach The Hospital Office, 428, Strand, at least one week before the date of each meeting.]

**The General Hospital, Coolgardie.**—This hospital had its origin in a private hospital founded in the very early days of the gold rush by two nurses who came from Adelaide. The Government, recognising that these ladies must live, and must be provided with means if they were to attend the poorer sick, gave a small subsidy. As the town grew, and illness with it, the accommodation was found to be totally inadequate. A public meeting was held, and, with the assent of the Government, the hospital was taken over by a local board of management. The Government gave £300 towards the erection of four suitable tents, and allowed £2 10s. for each patient unable to pay the hospital fees. As the hot weather approached the accommodation again proved inadequate, and the committee went to work, with the result that the patients were housed in huts. They are not imposing structures, but thanks to them and the staff employed much misery has been alleviated, and many persons have been restored to health. Especially was this the case during the height of the fever epidemic last February and March. We are informed the hospital death rate then was high; for many of the patients brought in were moribund. In the first six months of this year 535 indoor patients were admitted, and 237 outdoor cases were treated. Of this number 511 were discharged as cured and 24 as relieved, and there were 75 deaths, this large proportion being already explained. Why, it may be asked, appeal in London for aid to an hospital in Coolgardie? One good reason we have already given—that the work which broke down so many of these patients has made more people rich in London than in Coolgardie. Again, the control of the leading mining companies is in London, and it seems right that the directors and shareholders should be invited to subscribe towards an institution which does so much to maintain the health of Coolgardie and of those whose labour the companies require. Were the conditions of life on the goldfields normal, Government aid and local subscriptions would amply suffice to maintain the hospital, but the cost of hospital necessities is extremely high. With the growth of the population and the opening of still bigger and better accommodation, the expenditure must increase beyond the power of the locality to meet it. The receipts during the first six months of the year amount to £5,576, and expenditure to £5,506. This shows an apparent balance on the right side; but, unfortunately, the outstanding liabilities were fully £497. At the beginning of last month the debts were still over £400, and the resources of the hospital were practically exhausted. The particulars we have been enabled to give of this struggling and deserving institution were sent home in an official letter acknowledging a sum collected by Mr. A. H. P. Stoneham, who is so prominently associated with Western Australian enterprises, and we are glad to know that he is now moving to obtain further subscriptions. Contributions may be sent to the *Australian Mail*, 37, Walbrook, E.C.

